# Quality and safety innovations in the Radiology Department during the COVID-19 pandemic: a Latin American experience

**DOI:** 10.31744/einstein_journal/2020GS5832

**Published:** 2020-10-09

**Authors:** Patrícia Yokoo, Maria Carolina Bueno da Silva, Adham do Amaral e Castro, Eduardo Kaiser Ururahy Nunes Fonseca, Karine Minaif Martins, Marcos Roberto Gomes de Queiroz, Gilberto Szarf, Adriano Tachibana

**Affiliations:** 1 Hospital Israelita Albert Einstein São PauloSP Brazil Hospital Israelita Albert Einstein, São Paulo, SP, Brazil.

**Keywords:** Coronavirus, COVID-19, Coronavirus infections, Quality and safety, Tomography, X-ray computed

## Abstract

Radiology departments were forced to make significant changes in their routine during the coronavirus disease 2019 pandemic, to prevent further transmission of the coronavirus and optimize medical care as well. In this article, we describe our Radiology Department’s policies in a private hospital for coronavirus disease 2019 preparedness focusing on quality and safety for the patient submitted to imaging tests, the healthcare team involved in the exams, the requesting physician, and for other patients and hospital environment.

## INTRODUCTION

Recent cases of pneumonia of unknown cause in Wuhan, China, have led to the discovery of a new type of coronavirus, the severe acute respiratory syndrome coronavirus 2 (SARS-CoV-2). The disease is now known as coronavirus disease 2019 (COVID-19),^(1)^ and was declared a pandemic by the World Health Organization (WHO) on March 11, 2020.

The transmission of COVID-19 is through respiratory droplets and close contact, or in aerosol generating procedures,^(1)^ leading to a rapidly progressive spread of the virus.^(1-5)^ Local cases have been reported in almost all countries.^(1-5)^

Radiology plays an important role since chest computed tomography (CT) has proved to be a useful tool for evaluating the disease in the lungs, its extent, differential diagnoses and complications. Although its widespread use in screening is not recommended in a non-resource-constrained environment,^(6,7)^ it may also be used for suspected cases in different clinical scenarios.^(7)^

Chest radiography, although less sensitive,^(8)^ can be used for patient follow-up. The same applies to lung ultrasound, which emerged as an auxiliary tool for point-of-care follow-up of severe cases of COVID-19, in critical care units.^(9)^

Radiology departments embrace a wide range of activities and had to find some innovative solutions to face the demands of the pandemic, while maintaining their essential routine operations.^(10-12)^

Reorganizing processes in a radiology department to face a pandemic, considering quality and safety, has been a new challenge, and an organized and detailed description of the measures required was not available in the literature.

We describe our department policies for COVID-19 preparedness focusing on the necessary solutions, distributed into five main topics, as follows: preparing for the increased demand of imaging examinations; quality of care and safety of COVID-19 patients undergoing tests; safety of the healthcare team involved in examinations; the ordering physician; other patients and hospital environment.

### Background

Our Radiology Department is part of a not-for-profit private hospital. In 2018, the hospital had 579 beds and performed 5,131,194 diagnostic laboratory and imaging tests.

## PREPARING FOR THE INCREASED DEMAND OF IMAGING EXAMINATIONS

Initially, our main concern was the capacity to meet the increasing demand for CT ordered by the emergency department. Our current capacity for CT in this setting is 180 exams per day, performed in three different scanners (one dedicated to the emergency department and two shared with inpatient units). We predicted to reach full capacity within two months after our first COVID-19 case, when the peak of cases was expected. In order to be prepared, we decided to work in phases, in which a new CT scanner (a positron emission tomography/CT – PET/CT – and the single photon emission/CT – SPECT/CT) machine was located in the nuclear medicine area, and the devices of the outpatient’s clinic could be also be used (outpatients’ scheduled exams had been canceled at this point). A CT scan protocol was setup and staff were trained in all these locations anticipating their recruitment. By implementing these measures, our capacity would increase from 180 to 504 exams per day. Distant control capability was enabled in case of shortage of technicians due to infection, so that one professional could remotely operate the equipment, if necessary.

**Figure 1:**
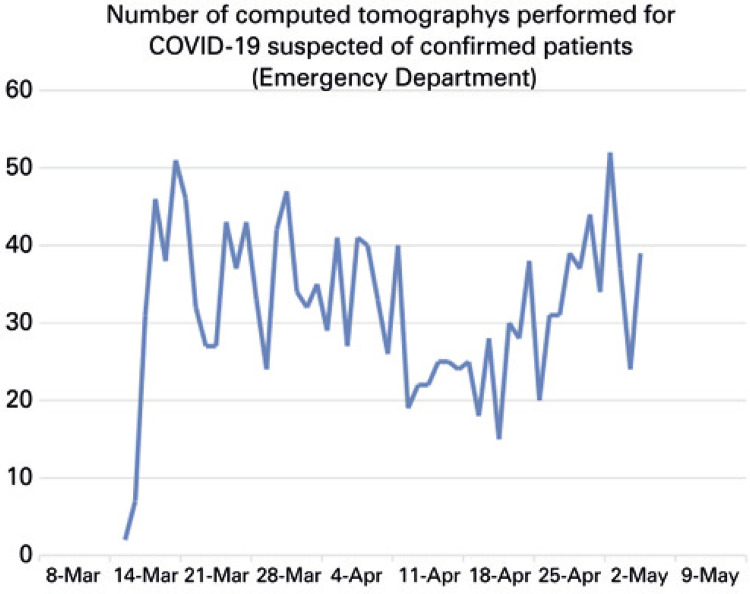
Total number of chest computed tomography exams ordered by the emergency department for confirmed or suspected coronavirus disease 2019 cases during the first weeks

After 2 months, our experience showed the number of chest CTs ordered by the emergency department for COVID-19 infection increased at a rate slower than what was initially projected. The peak of demand reached 51 tests per day (mean of 32), demanding less than 30% or our current capacity (Figure 1).

For hospitalized COVID-19 patients we initially predicted low usage of imaging resources, and that was confirmed during the first days of the pandemic. The initial experience showed one CT every 14 days of admission at the intensive care unit or stepdown unit, and one CT every 22 days of admission in inpatients units. Table 1 summarizes the CT usage for inpatients during the first two weeks of our experience.

**Table 1:** Imaging resource usage during the first 14 days of hospitalization of COVID-19 infected patients (93 patients)

Resource usage according to type of hospital admission	CT	X-ray	US	MRI	Interventional radiology
ICU					
Usage per day	0.06	0.73	0.07	0.01	0.01
Days of use	16.1	1.3	14.6	161	80.5
Probability of use (%)	28	100	39	6	6
Stepdown					
Usage per day	0.08	0.21	0	0	0
Days of use	13	4.9	0	0	0
Probability of use (%)	30	60	0	0	0
Inpatient unit					
Usage per day	0.08	0.09	0.01	0.01	0
Days of use	13.0	10.6	95.7	143.5	0
Probability of use (%)	26	17	3	2	0

CT: computed tomography; US: ultrasonography; MRI: magnetic resonance imaging; ICU: intensive care unit.

### Preparedness for performing outpatient exams

**Figure 2:**
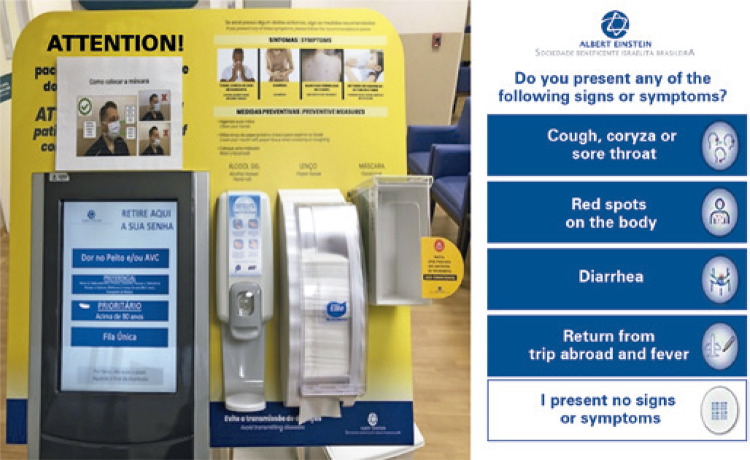
Electronic self-service queue ticket totem used to identify patients suspected of airway infections during COVID-19 pandemic. A question about signs or symptoms of epidemic diseases including COVID-19 infection was configured as a second page

**Figure 3:**
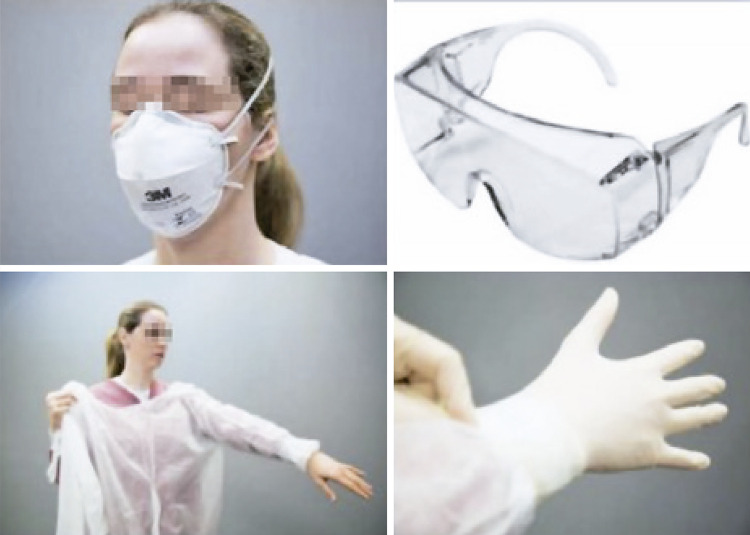
Instructions for the correct use of the personal protective equipment for contact, droplet and aerosol precautions, including safety glasses, N95 mask, gloves and gown. All healthcare professionals that take care of COVID-19 patients must comply

The most important task in performing outpatient imaging tests during the pandemic has been to identify suspected or positive patients as soon as possible, preferably before arriving at the radiology department, to apply the correct safety measures. That was accomplished by means of an electronic self-service queue token (Figure 2), which asked simple questions regarding signs or symptoms of respiratory infection. In case of positive answer, the administrative team was automatically activated, and the following procedures were implemented: a surgical mask was offered, hand hygiene was recommended and the nursing team was activated. The nurses wore personal protective equipment (PPE) and assessed the symptoms in more detail. When respiratory symptoms were identified, the team employed a series of precautions: to ensure the patient kept a surgical mask throughout the stay at the organization; to ensure a private space for the patient, apart from the general waiting room; to implement contact, droplet and aerosol precautions for the professional team involved in patient’s care, including the correct use of the PPEs, such as safety glasses, N95 mask, gloves and gown (Figure 3); at the end of the exam, the nursing staff, after changing gloves and gowns, cleaned the equipment and contact surfaces; if suspected or positive COVID-19 patients remained without a mask during the exam, and if an aerosol generating procedure was performed (in the radiology department the most relevant procedure is orotracheal intubation for tests under anesthesia), the exam room was isolated for two hours for passive air exchange and terminal cleaning. Scheduled procedures with expected aerosol generating procedures were preferably performed as the last exams of the day.

## RADIOLOGIST PREPAREDNESS

Although our Radiology Department counts with specialized thoracic radiologists, all medical staff were trained to recognize the typical imaging findings for COVID-19 pneumonia, and a set of confirmed case images were provided for review and self-assessment. A structured report was developed to optimize the workflow (Appendix 1).^(13)^

To improve knowledge about the typical radiological findings of COVID-19, online lectures were prepared by thoracic radiologists using digital platforms, including question and answer sessions. Classes were presented online, to avoid crowding. These education innovations are necessary in pandemic scenarios, as observed in past outbreak experiences.^(14)^

The number of radiologists working at the hospital was reduced and home office was implemented. All educational and administrative meetings were adapted to be held remotely. Special care was taken to provide home office for the staff over 60 years of age, and for employees with health conditions that could be a risk factor for critical COVID-19 infection.

**Figure 4:**
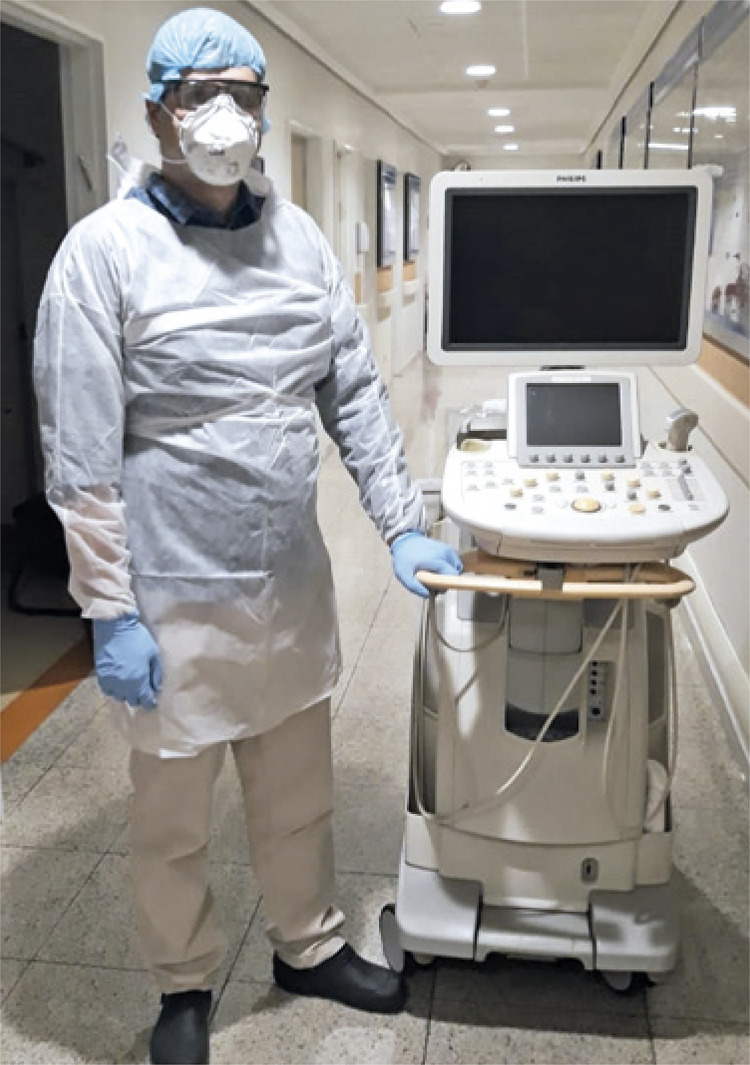
Radiologists perform all the ultrasound examinations in our institution, and were instructed to carried out exams of the COVID-19 patients at bedside. Personal protective equipment, such as gloves, gowns, face mask, and safety glasses, with the respective training of usage was made available to all involved

Ultrasound of COVID-19 patients were carried out on the bedside to avoid their transportation. All staff involved, including radiologists who perform all ultrasound examinations at our organization, received specific training for donning and doffing and use of appropriate PPEs. All COVID-19 patients were allocated in negative pressure rooms and were oriented to use surgical masks at all times (Figure 4).

## THE ORDERING PHYSICIAN

The imaging modality of choice to assess COVID-19 patients is chest CT.^(7)^ Ordering physicians were instructed to request CT only for patients with severe symptoms and requiring hospitalization. Clinical signs and symptoms for indicating CT comprise oxygen saturation <93%, respiratory rate >24 breaths per minute, as well as dyspnea and abnormal lung auscultation.

To facilitate communication, our department provided a telephone list for direct contact with each radiologic specialty, available 24 hours/seven days a week. Ordering physicians were oriented to no longer visit the reporting room for case reviews, thus avoiding possible contamination of the room and radiology staff. Screen sharing and Web conferencing were strategies adopted for case discussion. Audiovisual reports sent by messenger applications were another strategy used to enhance the clinical discussions with no in-person meetings.

The thoracic radiology staff prepared an assertive structured report focused on the pandemic (Appendix 1). Of special note, the subjective evaluation of lung parenchyma involvement of more or less than 50% was included, since our physicians considered this piece of information important in the algorithm for decision making regarding hospitalization.^(15-17)^

## IMPROVEMENTS IN RADIOLOGY REPORTING WORKFLOW

We created a special flag in our reporting system to prioritize imaging studies performed with the clinical indication of suspected or confirmed COVID-19, aiming to optimize report release and streamline decision-making process.

Also, an unexpected finding suggestive of COVID-19 infection in chest CT ordered for other clinical indications is considered a critical finding, and follows the same algorithm created to meet the Joint Commission International quality and safety standards, since our organization holds its accreditation. Technicians were trained to recognize suspicious findings on chest CT and promptly call radiologists.

All radiologists were also oriented to contact ordering physicians as soon as possible, within 1 hour. The same process applies to other CT exams that incidentally find signs of COVID infection (*e.g*. abdominal or spine CT).

## HOSPITAL ENVIRONMENT

We created different paths throughout the organization to receive patients testing positive (or suspected) or negative for COVID-19, who were submitted to imaging tests, using almost exclusive staff, equipment and waiting rooms for each group of patients. Extreme care was taken to perform imaging tests in separate location for inpatients to prevent hospital-acquired COVID-19 infection. Now the peak of infectious cases is passing, providing a safe and reliable environment is crucial for patients who require exams for indications other than COVID-19-related evaluation.

Like ultrasound, almost all radiographs are performed on the bedside. The same decontamination protocols must be used after examining patients infected with coronavirus, and PPEs are crucial. Specific ultrasound machines and portable X-ray machines were assigned for exclusive use in COVID-19 patients (Figure 4).

## OTHER PREPAREDNESS MEASURES

To ensure safety of patients who need hospital care in this pandemic, the organization adopted some measures to reduce patient visits, and postpone outpatient examinations and surgical procedures. Medical consultations by telemedicine were encouraged.

The hospital offers a special clinic for all health professionals involved in care of COVID-19 patients, who present respiratory symptoms, with rapid testing and isolation, whenever necessary. It is important to note that in addition to therapeutic measures to treat positive cases, psychological support is available to all those engaged in the fight against COVID-19. Keeping communication channels open with the leadership is very important to identify staff under stress.

Since there is community transmission of the infection, all staff, including administrative team, were advised to use a surgical mask to avoid transmission among workers and patients. Additionally, the temperature of every worker is measured upon arrival to work, by means of a forehead thermometer or a fully automatic artificial intelligence assisted facial thermal imaging (developed in-house).

Table 2 summarizes the recommendations for radiology department preparedness.

**Table 2:** Summary of the recommended procedures by type of preparedness

Type of preparedness	Procedure
Increase in volume of exams	Plan in advance to use all resources available
	Train all possible staff to be prepared to perform the most often demanded exams
	Try to predict radiology department usage and be prepared
	Use remote access to perform exams remotely
Outpatient exams	To ensure an effective triage of COVID-19 positive or suspected patients and establish droplet precautions
	Make sure the staff wear PPEs
	Comply with room cleaning procedures after the examination (aerosol and/or droplet precautions)
	Use an electronic self-service queue token
Radiologists	Train all radiologists to recognize typical COVID-19 findings
	Encourage home office
	Train on use of PPEs
Ordering physician	Provide appropriateness criteria for decision-making support: X-ray as first-line imaging modality; CT for severe patients and to estimate illness severity and help decide about hospitalization
	Design a structured report to improve understanding
	Maintain communication over the phone or by messenger applications
	Use audiovisual reports
Reporting workflow	Flag exams in the radiology information system
	Incidental diagnosis of COVID-19 infection considered as a “critical finding” (must communicate result over the phone)
Hospital environment	Perform X-ray and ultrasound on the bedside
	Create separate paths for positive and negative COVID-19 patients to avoid hospital-acquired infection, and to provide safety to patients that cannot postpone their examinations
Other measures	Test and isolate positive healthcare providers
	Temperature measurement for all healthcare providers upon arrival at work
	Use of surgical masks by all workers
	Postponing outpatient consultation
	Enhance the use of Telemedicine
	Keep communication opened

PPE: personal protective equipment; CT: computed tomography;COVID-19: coronavirus disease 2019.

## DISCUSSION

The diagnostic process of individuals with COVID-19 directly involves the radiology department. In the context of a highly contagious disease, measures to protect the healthcare team and patients must be taken.

In addition to minimizing the potential risks of contamination of contacts, rapid diagnosis can be decisive for prognosis. Tan et al. had suggested labeling the suspected COVID-19 studies with ‘high alert’ for easy identification and rapid communication of results.^(18)^

Some studies have already recognized the effect of complications of healthcare-associated infections, including increased morbidity and mortality of patients and healthcare staff, longer length of stay, and requiring additional diagnostic and therapeutic interventions.^(19,20)^

Of note, this organization protocol is dynamic and has been improved daily, as new information about COVID-19 becomes available. We hope this pandemic context raises awareness for the adoption of continuous infection control practices.

## CONCLUSION

We detailed the quality and safety innovations of our Radiology Department to meet the demands of a new reality during a pandemic crisis. Our aim is to assure quality of health services provided and safety of our patients and employees. Describing our experience, we expect to provide useful information and examples of innovations that can help other radiology departments. Some of the newly applied measures will certainly remain in practice once the outbreak is over.

## Appendix 1

**Table:** A structured report was developed to optimize workflow

High-resolution computed tomography of the lungs
**Technique**
Multislice helical CT, no intravenous contrast.
**Indication**
Evaluation of pulmonary infectious process (investigation of pulmonary involvement by COVID-19).
**Analysis**
[COVID-19 positive] Multiple ground-glass pulmonary opacities, sometimes associated to interlobular septal thickening, fine reticulation (in addition to sparse consolidation foci), presenting bilateral multifocal distribution, mostly peripheral and posterior, and more extensively in the lower lobes. Although not specific, such findings are consistent with viral pneumonia, and the possibility of COVID-19 should be considered among the differential diagnoses.
[Severity estimate] The estimated extent of lung involvement on tomography is (lesser / greater) than 50% (visual analysis).
[Pneumonia of unknown etiology] (Patterns that are neither typical nor completely atypical of COVID-19). These findings are not specific, but they usually represent a pulmonary inflammatory/infectious disease; COVID-19 could be included among differential diagnoses.
[Lobar pneumonia, bronchopneumonia, infectious bronchiolitis] Such findings are compatible with the pulmonary infectious process, whose characteristics are not commonly found in cases of lung involvement by COVID-19; other etiologic agents should be initially considered for differential diagnoses.
[No sign of infection] Absence of focal pulmonary opacities suggestive of an active parenchymal infection. It should be noted that the absence of tomographic signs of pneumonia in the first days after onset of symptoms does not rule out COVID-19.
**Other findings**
Absence of pleural effusion.
Remaining lung parenchyma with no significant changes.
No mediastinal lymphadenopathy.
Other thoracic structures with no relevant changes in the clinical context.

CT: computed tomography; COVID-19: coronavirus disease 2019.L
