# Supplementary material for: Quality and safety innovations in the Radiology Department during the COVID-19 pandemic: a Latin American experience
Source: Einstein (Sao Paulo). 2020 Oct 9;18:eGS5832. doi: 10.31744/einstein_journal/2020GS5832 (PMC7546680; doi:10.31744/einstein_journal/2020GS5832)
Supplement: Translation

# Translation: Inovações de qualidade e segurança no Departamento de Radiologia durante a pandemia pela COVID-19: uma experiência Latino-Americano

**Authors:** Patrícia Yokoo, Maria Carolina Bueno da Silva, Adham do Amaral e Castro, Eduardo Kaiser Ururahy Nunes Fonseca, Karine Minaif Martins, Marcos Roberto Gomes de Queiroz, Gilberto Szarf, Adriano Tachibana

**Affiliations:** 1 Brasil Hospital Israelita Albert Einstein, São Paulo, SP, Brasil.

**Keywords:** Coronavírus, COVID-19, Infecções por coronavírus, Qualidade e segurança, Tomografia computadorizada por raios X

## Abstract

Os departamentos de radiologia precisaram adotar mudanças significativas em sua rotina durante a pandemia da doença causada pelo novo coronavírus, a fim de reduzir sua transmissibilidade e otimizar os cuidados médicos. Neste artigo, descrevemos as políticas adotadas pelo Departamento de Radiologia de um hospital privado durante a pandemia, com foco em qualidade e segurança de paciente submetido a exames de imagem, equipe de assistência do departamento de imagem, médico solicitante, demais pacientes e ambiente hospitalar.

## INTRODUÇÃO

Casos recentes de pneumonia de causa desconhecida em Wuhan, China, levaram à descoberta de um novo tipo de coronavírus, o coronavírus da síndrome respiratória aguda grave 2 (SARS-CoV-2). A doença é conhecida como doença do novo coronavírus 2019 (COVID-19)^(1)^ e foi declarada uma pandemia pela Organização Mundial da Saúde (OMS) em 11 de março de 2020.

A transmissão da COVID-19 dá-se por meio de gotículas respiratórias e contato próximo, ou em procedimentos com geração de aerossóis.^(1-5)^ Já foram relatados casos locais em quase todos os países.^(1-5)^

A radiologia desempenha papel importante nessa pandemia, já que a tomografia computadorizada (TC) de tórax é uma ferramenta comprovadamente útil para avaliar o efeito da doença nos pulmões, incluindo extensão, diagnósticos diferenciais e complicações. Embora o uso indiscriminado para rastreamento não seja recomendado em contextos sem restrição de recursos,^(6,7)^ essa modalidade também pode ser usada para casos suspeitos em diferentes cenários clínicos.^(7)^

A radiografia de tórax, embora menos sensível,^(8)^ pode ser usada no acompanhamento dos pacientes. O mesmo se aplica à ultrassonografia dos pulmões, que surgiu como uma ferramenta auxiliar para acompanhamento à beira do leito de casos graves de COVID-19, em unidades de cuidados intensivos.^(9)^

Os departamentos de radiologia desempenham uma gama de atividades e tiveram que encontrar soluções inovadoras para atender às demandas da pandemia e manter, ao mesmo tempo, suas operações de rotina essenciais.^(10-12)^

A reorganização dos processos em um departamento de radiologia para enfrentar a pandemia, garantindo a qualidade dos serviços e a segurança dos pacientes, é um novo desafio. Não existe ainda, na literatura, uma descrição organizada e detalhada das medidas necessárias para esse fim.

Descrevemos aqui as políticas de nosso departamento relativas à preparação para a COVID-19, com foco nas soluções necessárias, distribuídas em cinco tópicos principais, a saber: preparação para o aumento da demanda por exames de imagem; qualidade do atendimento e segurança dos pacientes com COVID-19 submetidos a exames; segurança da equipe de saúde envolvida nos exames; médico solicitante; outros pacientes e o ambiente hospitalar.

### Sobre o departamento

Nosso Departamento de Radiologia faz parte de um hospital privado, sem fins lucrativos. Em 2018, o hospital tinha 579 leitos e realizou 5.131.194 exames diagnósticos laboratoriais e de imagem.

## PREPARAÇÃO PARA O AUMENTO DA DEMANDA POR EXAMES DE IMAGEM

Inicialmente, nossa principal preocupação era a capacidade de atender à crescente demanda por TCs solicitadas pelo setor de emergência. Nossa capacidade, nesse contexto, era de 180 exames de TC por dia, realizados em três *scanners* diferentes (um dedicado ao setor de emergência e dois compartilhados com as unidades de internação). Nossa previsão era de que atingiríamos nossa capacidade máxima 2 meses após nosso primeiro caso de COVID-19, quando esperávamos que ocorresse o pico no número de casos. Para nos prepararmos, decidimos trabalhar em fases, sendo que um novo *scanner* de TC (uma máquina de TC por emissão de pósitrons – PET/CT – e de TC por emissão de fóton único – SPECT/CT) foi colocado na área de medicina nuclear, e os equipamentos do ambulatório também poderiam ser usados (os exames ambulatoriais agendados já haviam sido cancelados a essa altura). Um protocolo para exames de TC foi definido, e os funcionários foram treinados em todos esses locais, prevendo-se que seriam recrutados. Essas medidas permitiriam aumentar nossa capacidade de 180 para 504 exames por dia. Habilitamos a capacidade de controle à distância, para o caso de falta de contingente devido à infecção, para que um único profissional pudesse operar os equipamentos remotamente, se necessário.

Após 2 meses, nossa experiência mostrou que o número de TC de tórax solicitadas pelo setor de emergência devido à infecção por COVID-19 aumentou a uma taxa mais lenta do que a inicialmente prevista. O pico de demanda foi atingido aos 51 exames por dia (média de 32), ou seja, menos de 30% ou nossa capacidade atual (Figura 1).

**Figura 1:** Número total de exames de tomografia computadorizada do tórax solicitado pelo setor de emergência para casos confirmados ou suspeitos de COVID-19 durante as primeiras semanas

Para pacientes de COVID-19 hospitalizados, previmos, inicialmente, um baixo uso de recursos de imagem, o que foi confirmado durante os primeiros dias da pandemia. A experiência inicial mostrou uma TC a cada 14 dias de internação na unidade de terapia intensiva ou semi-intensiva e uma TC a cada 22 dias nas unidades de internação. A tabela 1 resume o uso de TC para pacientes internados durante as duas primeiras semanas de nossa experiência.

**Tabela 1:** Uso de recursos de imagem nos primeiros 14 dias de hospitalização para pacientes como COVID-19 (93 pacientes)

Uso de recursos por tipo de internação hospitalar	TC	Radiografia	US	RM	Radiologia interverncionista
UTI					
Uso por dia	0,06	0,73	0,07	0,01	0,01
Dias de uso	16,1	1,3	14,6	161	80,5
Probabilidade de uso (%)	28	100	39	6	6
Semi-intensiva					
Uso por dia	0,08	0,21	0	0	0
Dias de uso	13	4,9	0	0	0
Probabilidade de uso (%)	30	60	0	0	0
Unidade de internação					
Uso por dia	0,08	0,09	0,01	0,01	0
Dias de uso	13,0	10,6	95,7	143,5	0
Probabilidade de uso (%)	26	17	3	2	0

TC: tomografia computadorizada; US: ultrassonografia; RM: ressonância magnética; UTI: unidade de terapia intensiva.

## PREPARAÇÃO PARA A REALIZAÇÃO DE EXAMES AMBULATORIAIS

A tarefa mais importante na realização de exames ambulatoriais durante a pandemia foi identificar pacientes com suspeita ou confirmação de infecção assim que possível, de preferência antes que chegassem ao departamento de radiologia, para implementação das medidas de segurança corretas. Isso foi feito por meio de um sistema de autoatendimento eletrônico para retirada de senhas (Figura 2), que fazia perguntas simples quanto a sinais ou sintomas de infecção respiratória. Em caso de respostas afirmativas, a equipe administrativa era automaticamente acionada, e os procedimentos a seguir eram implementados: fornecimento de máscara cirúrgica, recomendação de higienização das mãos e acionamento da equipe de enfermagem. Os enfermeiros tinham que usar equipamentos de proteção individual (EPIs) e avaliar os sintomas com mais detalhes. Caso houvesse sintomas respiratórios presentes, a equipe tinha que empregar uma série de precauções: garantir que o paciente usasse máscara cirúrgica durante toda a permanência na organização; colocar o paciente em um espaço reservado, separado da sala de espera do público em geral; implementar precauções de contato, gotículas e aerossóis para a equipe profissional envolvida no atendimento ao paciente, incluindo uso correto de EPIs, como óculos de segurança, máscara N95, luvas e avental (Figura 3); no fim do exame, a equipe de enfermagem, após trocar as luvas e o avental, limpava o equipamento e as superfícies de contato; se um caso suspeito ou confirmado de COVID-19 realizasse um exame sem máscara, e se um procedimento com geração de aerossóis fosse realizado (no departamento de radiologia, o procedimento mais relevante é intubação orotraqueal para exames sob anestesia), a sala de exame era isolada por 2 horas para troca passiva de ar e limpeza terminal. Exames com possíveis procedimentos geradores de aerossóis eram agendados nos últimos horários do dia.

**Figura 2:** Totem de autoatendimento eletrônico para retirada de senhas, com identificação dos pacientes com suspeita de infecção de vias aéreas durante a pandemia pela COVID-19. Uma pergunta sobre sinais ou sintomas de doenças epidêmicas, inclusive COVID-19, foi configurada como uma segunda página

**Figura 3:** Instruções para o uso correto dos equipamentos de proteção individual para precauções de contato, gotículas e aerossóis, incluindo óculos de segurança, máscara N95, luvas e avental. Todos os profissionais de saúde que atendam pacientes com COVID-19 devem cumprir as exigências

## PREPARAÇÃO DO RADIOLOGISTA

Embora nosso Departamento de Radiologia conte com especialistas em radiologia torácica, todos os médicos foram treinados para saber reconhecer achados de imagem típicos da pneumonia da COVID-19, e uma série de imagens de casos confirmados foi disponibilizada para análise e autoavaliação. Um laudo estruturado foi elaborado para otimização do fluxo de trabalho (Apêndice 1).^(13)^

Para melhorar os conhecimentos sobre os achados radiológicos típicos da COVID-19, aulas online foram preparadas por especialistas em radiologia torácica usando plataformas digitais, incluindo sessões de perguntas e respostas. As aulas foram apresentadas pela Internet, para evitar aglomerações. Essas inovações na área do ensino são necessárias no contexto de uma pandemia, conforme observado em experiências prévias com surtos de doenças.^(14)^

O número de radiologistas atuando no hospital foi reduzido, tendo sido implementado trabalho remoto. Todas as reuniões educacionais e administrativas foram adaptadas para serem realizadas remotamente. Esforços especiais foram empregados para viabilizar o trabalho remoto de funcionários acima de 60 anos de idade ou com doenças preexistentes, que pudessem ser fatores de risco para uma pior evolução da COVID-19.

Os exames de ultrassonografia de pacientes de COVID-19 foram realizados à beira do leito, para evitar deslocamentos. Todos os profissionais envolvidos, incluindo os radiologistas encarregados de realizar exames de ultrassonografia em nossa organização, receberam treinamento específico sobre paramentação, desparamentação e uso correto de EPIs. Todos os pacientes de COVID-19 foram alocados em salas com pressão negativa e orientados a usar máscaras cirúrgicas o tempo todo (Figura 4).

**Figura 4:** Os radiologistas realizam todos os exames de ultrassonografia em nossa instituição, e foram instruídos a realizar exames em pacientes com COVID-19 à beira do leito. Equipamentos de proteção individual, como luvas, avental, máscara facial e óculos de segurança, foram disponibilizados a todos os envolvidos, assim como treinamento para o uso deles

## MÉDICO SOLICITANTE

A modalidade de imagem de escolha para avaliação de pacientes com COVID-19 é a TC de tórax.^(7)^Os médicos solicitantes foram instruídos a solicitar TC somente para pacientes com sintomas graves e necessidade de hospitalização. Os sinais e os sintomas para indicação de TC incluem saturação de oxigênio <93%, frequência respiratória >24 respirações por minuto, dispneia e alterações à ausculta pulmonar.

Para facilitar a comunicação, nosso departamento forneceu uma lista telefônica para contato direto com cada especialidade radiológica, disponível 24 horas, 7 dias por semana. Os médicos solicitantes foram orientados a não mais se dirigir à sala de laudos para analisar os casos, evitando a possibilidade de contaminação da sala e da equipe de radiologia. As estratégias adotadas para discussão de casos foram compartilhamento de tela e conferência via Web. Relatórios audiovisuais enviados por aplicativos de mensagens foram outra estratégia usada para aprimorar as discussões clínicas sem reuniões presenciais.

A equipe de radiologia torácica preparou um laudo estruturado e assertivo, com foco na pandemia (Apêndice 1). É importante ressaltar que foi incluída uma avaliação subjetiva de acometimento superior ou inferior a 50% do parênquima pulmonar, já que nossos médicos consideraram essa informação importante no algoritmo de tomada de decisão relativa à hospitalização.^(15-17)^

## MELHORIAS NO FLUXO DE TRABALHO DA LIBERAÇÃO DE LAUDOS DE RADIOLOGIA

Criamos um sinalizador especial em nosso sistema para priorizar estudos de imagens realizados com indicação clínica de suspeita ou confirmação de COVID-19, a fim de otimizar a liberação de laudos e agilizar a tomada de decisão.

Além disso, achados inesperados sugestivos de COVID-19 em TC de tórax solicitadas com outras indicações clínicas são considerados críticos e seguem o mesmo algoritmo criado para atender aos padrões de qualidade e segurança da *Joint Commission International*, já que nossa organização é certificada. Os técnicos foram treinados para reconhecer achados suspeitos em TC de tórax e entrar em contato imediatamente com os radiologistas.

Todos os radiologistas também foram orientados a entrar em contato com o médico solicitante assim que possível, em, no máximo, 1 hora. O mesmo processo se aplica a outros exames de TC que incidentalmente revelem sinais de infecção pelo novo coronavírus (por exemplo: TC abdominal ou de coluna).

## AMBIENTE HOSPITALAR

Criamos rotas diferentes em toda a organização para receber pacientes com resultado positivo (ou suspeita) ou negativo para COVID-19, que precisassem ser submetidos a exames de imagem, usando equipes, equipamentos e salas de espera praticamente exclusivas para cada grupo de pacientes. Foi tomado extremo cuidado para a realização de exames de imagem em locais separados para pacientes internados, para prevenir o risco de infecção hospitalar pela COVID-19. Com a diminuição do pico de casos infecciosos, proporcionar um ambiente seguro e confiável é crucial para pacientes que necessitam de exames com indicações não relacionadas à COVID-19.

Assim como os exames de ultrassonografia, quase todas as radiografias são realizadas à beira do leito. Os mesmos protocolos de descontaminação devem ser usados após a realização de exames em pacientes infectados com o novo coronavírus, e o uso de EPIs é indispensável. Máquinas de ultrassonografia específicas e equipamentos portáteis de raio X foram reservados para uso exclusivo em pacientes com COVID-19 (Figura 4).

## OUTRAS MEDIDAS

Para assegurar a segurança dos pacientes que precisam de atendimento hospitalar durante a pandemia, a organização adotou algumas medidas para reduzir as visitas de pacientes e adiar exames ambulatoriais e procedimentos cirúrgicos. Foi incentivado o uso da telemedicina para consultas médicas.

O hospital oferece uma clínica especial para todos os profissionais de saúde envolvidos no atendimento a pacientes com COVID-19 que apresentam sintomas respiratórios, com testagem rápida e isolamento, quando necessário. É importante observar que, além das medidas terapêuticas para tratar casos positivos, há suporte psicológico disponível para todos os envolvidos no combate à COVID-19. Manter os canais de comunicação abertos com a liderança é muito importante para a identificação de funcionários que estejam sob estresse.

Como há transmissão comunitária da infecção, todos os funcionários, inclusive a equipe administrativa, foram instruídos a usar máscara cirúrgica para evitar a transmissão entre trabalhadores e pacientes. Além disso, a temperatura de todos os trabalhadores é medida na chegada ao trabalho, usando-se um termômetro sem contato ou um sistema de imagem térmica facial assistida por inteligência artificial, totalmente automático (desenvolvido internamente).

A tabela 2 resume as recomendações para preparo do departamento de radiologia.

**Tabela 2:** Resumo dos procedimentos recomendados por tipo de preparo

Tipo de preparo	Procedimento
Aumento do volume de exames	Planejar com antecedência para usar todos os recursos disponíveis
	Treinar todos os funcionários possíveis na realização dos exames mais frequentemente solicitados
	Tentar prever o uso do departamento de radiologia e garantir que tudo esteja preparado
	Usar acesso remoto para realizar exames
Exames ambulatoriais	Assegurar triagem efetiva de pacientes com suspeita ou confirmação de COVID-19 e implementar precauções de gotículas
	Assegurar que os funcionários estejam usando EPIs
	Cumprir os procedimentos de limpeza da sala após exames (precauções para aerossóis e/ou gotículas)
	Usar um sistema de autoatendimento eletrônico para retirada de senhas
Radiologistas	Treinar todos os radiologistas para reconhecer achados típicos da COVID-19
	Incentivar o trabalho remoto
	Treinar quanto ao uso de EPIs
Médico solicitante	Fornecer critérios para embasar a tomada de decisão: radiografia como exame de imagem de primeira linha; TC para pacientes graves e para estimar a gravidade da doença e ajudar a decidir sobre necessidade de hospitalização
	Elaborar um laudo estruturado para melhorar a compreensão
	Manter comunicação pelo telefone ou por aplicativos de mensagens
	Usar relatórios audiovisuais
Fluxo de trabalho para emissão de laudos	Sinalizar exames no sistema de informações de radiologia
	Diagnóstico incidental de COVID-19 considerado como “achado crítico” (o resultado deve ser informado por telefone)
Ambiente hospitalar	Realizar exames de radiografia e ultrassonografia à beira do leito
	Criar rotas separadas para pacientes positivos e negativos para COVID-19, para evitar infecções hospitalares e garantir a segurança de pacientes que não puderem adiar seus exames
Outras medidas	Testar e isolar profissionais de saúde com resultado positivo
	Medir temperatura para todos os profissionais de saúde no momento da chegada ao trabalho
	Uso de máscaras cirúrgicas por todos os trabalhadores
	Adiamento de consultas ambulatoriais
	Avançar no uso da telemedicina
	Manter a comunicação aberta

EPI: equipamentos de proteção individual; TC: tomografia computadorizada; COVID-19: doença causada pelo novo coronavírus 2019.

## DISCUSSÃO

O processo de diagnóstico de indivíduos com a COVID-19 envolve diretamente o departamento de radiologia. No contexto de uma doença altamente contagiosa, deve-se tomar medidas para proteger a equipe de saúde e os pacientes.

Além de minimizar os riscos potenciais de contaminação de contatos, o diagnóstico rápido pode ser decisivo para o prognóstico. Tan et al. sugeriram a sinalização dos estudos de casos suspeitos de COVID-19 com alerta máximo para uma fácil identificação e rápida liberação de resultados.^(18)^

Alguns estudos já reconheceram o efeito de complicações de infecções associadas aos cuidados de saúde, incluindo aumento da morbidade e mortalidade de pacientes e profissionais de saúde, tempos de internação mais longos e necessidade de intervenções diagnósticas e terapêuticas adicionais.^(19,20)^

É importante destacar que este protocolo organizacional é dinâmico e tem sido aprimorado diariamente, conforme novas informações sobre a COVID-19 são disponibilizadas. Esperamos que o contexto desta pandemia possa conscientizar sobre a adoção de práticas contínuas de controle de infecções.

## CONCLUSÃO

Detalhamos as inovações de qualidade e segurança em nosso Departamento de Radiologia para atender a demandas de uma nova realidade durante a pandemia. Nosso objetivo é garantir a qualidade dos serviços de saúde prestados e a segurança de nossos pacientes e funcionários. Ao descrever nossa experiência, pretendemos oferecer informações úteis e exemplos de inovações que possam ajudar outros departamentos de radiologia. Algumas das novas medidas implementadas certamente continuarão sendo usadas depois da pandemia.

## Apêndice 1

**Table:** Laudo estruturado desenvolvido para otimizar o fluxo de trabalho

Tomografia computadorizada de alta resolução dos pulmões
**Técnica**
Tomografia computadorizada helicoidal *multislice*, sem contraste intravenoso.
**Indicação**
Avaliação de processo infeccioso pulmonar (investigação de acometimento pulmonar pela COVID-19).
**Análise**
[Resultado positivo para COVID-19] Múltiplas opacidades pulmonares em vidro fosco, por vezes associadas a espessamento septal interlobular, reticulado fino (além de focos esparsos de consolidação), apresentando distribuição multifocal bilateral, predominantemente periférica e posterior, e mais extensa nos lobos inferiores. Embora não sejam específicos, esses achados são típicos de pneumonia viral, e a possibilidade de COVID-19 deve ser considerada entre os diagnósticos diferenciais.
[Estimativa de gravidade] A estimativa da extensão do acometimento pulmonar na tomografia é (inferior/superior) a 50% (análise visual).
[Pneumonia de etiologia desconhecida] (Padrões indeterminados para COVID-19). Esses achados não são específicos, mas geralmente representam doença pulmonar inflamatória/infecciosa; a COVID-19 pode ser incluída nos diagnósticos diferenciais.
[Pneumonia lobar, broncopneumonia, bronquiolite infecciosa] Estes achados são compatíveis com processo infeccioso pulmonar, cujas características não são frequentemente encontradas em caso de acometimento pulmonar pela COVID-19; outros agentes etiológicos devem ser inicialmente considerados para diagnósticos diferenciais.
[Sem sinal de infecção] Ausência de opacidades pulmonares focais sugestivas de infecção parenquimatosa ativa. Deve-se ressaltar que a ausência de sinais tomográficos de pneumonia nos primeiros dias após o início dos sintomas não elimina a hipótese de COVID-19.
**Outros achados**
Ausência de derrame pleural.
Parênquima pulmonar remanescente sem alterações significativas.
Sem linfadenopatia mediastinal.
Outras estruturas torácicas sem alterações relevantes no contexto clínico.

COVID-19: doença causada pelo novo coronavírus 2019.
